# Ethyl 1-*sec*-butyl-2-(2-hydroxy­phen­yl)-1*H*-benzimidazole-5-carboxyl­ate 0.25-hydrate

**DOI:** 10.1107/S1600536810015448

**Published:** 2010-05-08

**Authors:** Natarajan Arumugam, Aisyah Saad Abdul Rahim, Hasnah Osman, Madhukar Hemamalini, Hoong-Kun Fun

**Affiliations:** a School of Pharmaceutical Sciences, Universiti Sains Malaysia, 11800 USM, Penang, Malaysia; b School of Chemical Sciences, Universiti Sains Malaysia, 11800 USM, Penang, Malaysia; c X-ray Crystallography Unit, School of Physics, Universiti Sains Malaysia, 11800 USM, Penang, Malaysia

## Abstract

In the title compound, C_20_H_22_N_2_O_3_·0.25H_2_O, the water mol­ecule (occupancy 0.25) is disordered across a crystallographic inversion center. The dihedral angle between the hydroxy­phenyl ring and the benzimidazole ring system is 59.31 (9)°. In the crystal structure, mol­ecules are connected by inter­molecular O—H⋯N and C—H⋯O hydrogen bonds. The crystal structure is further stabilized by a weak C—H⋯π inter­action involving the imidazole ring.

## Related literature

For background to benzimidazoles and their biological importance, see: Garuti *et al.* (2004 ▶); Bonfanti *et al.* (2008 ▶); Ozden *et al.* (2008 ▶); Shao *et al.* (2005 ▶); Blythin *et al.* (1986 ▶); Snow (2007 ▶). For the synthesis of benzimidazoles, see: Arumugam *et al.* (2010**a* ▶,*b* ▶,c* ▶). For the stability of the temperature controller used in the data collection, see: Cosier & Glazer (1986 ▶).
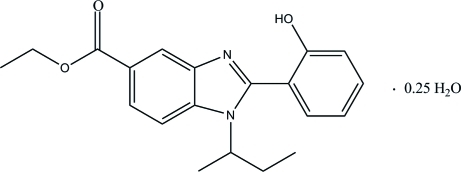


## Experimental

### 

#### Crystal data

C_20_H_22_N_2_O_3_·0.25H_2_O
*M* *_r_* = 342.90
Monoclinic, 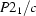

*a* = 7.0484 (11) Å
*b* = 27.262 (4) Å
*c* = 9.4673 (14) Å
β = 97.495 (3)°
*V* = 1803.6 (5) Å^3^
*Z* = 4
Mo *K*α radiation
μ = 0.09 mm^−1^
*T* = 100 K
0.34 × 0.21 × 0.05 mm

#### Data collection

Bruker APEX DUO CCD area-detector diffractometer
Absorption correction: multi-scan (*SADABS*; Bruker, 2009 ▶) *T*_min_ = 0.971, *T*_max_ = 0.996
18277 measured reflections
4738 independent reflections
3152 reflections with *I* > 2σ(*I*)
*R*_int_ = 0.064

#### Refinement

*R*[*F* ^2^ > 2σ(*F* ^2^)] = 0.066
*wR*(*F* ^2^) = 0.232
*S* = 1.08
4738 reflections
243 parameters
H atoms treated by a mixture of independent and constrained refinement
Δρ_max_ = 0.35 e Å^−3^
Δρ_min_ = −0.46 e Å^−3^

### 

Data collection: *APEX2* (Bruker, 2009 ▶); cell refinement: *SAINT* (Bruker, 2009 ▶); data reduction: *SAINT*; program(s) used to solve structure: *SHELXTL* (Sheldrick, 2008 ▶); program(s) used to refine structure: *SHELXTL*; molecular graphics: *SHELXTL*; software used to prepare material for publication: *SHELXTL* and *PLATON* (Spek, 2009 ▶).

## Supplementary Material

Crystal structure: contains datablocks global, I. DOI: 10.1107/S1600536810015448/sj2770sup1.cif

Structure factors: contains datablocks I. DOI: 10.1107/S1600536810015448/sj2770Isup2.hkl

Additional supplementary materials:  crystallographic information; 3D view; checkCIF report

## Figures and Tables

**Table 1:** Hydrogen-bond geometry (Å, °) *Cg*1 is the centroid of the N1,N2,C1,C2,N7 imidazole ring.

*D*—H⋯*A*	*D*—H	H⋯*A*	*D*⋯*A*	*D*—H⋯*A*
O1—H1*O*1⋯N1^i^	0.96 (4)	1.75 (4)	2.691 (3)	168 (3)
C14—H14*C*⋯O1^i^	0.96	2.45	3.398 (3)	168
C17—H17*A*⋯*Cg*1^ii^	0.93	2.96	3.734 (3)	142

Symmetry codes: (i) 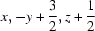; (ii) 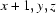.

**Fig. 1.**
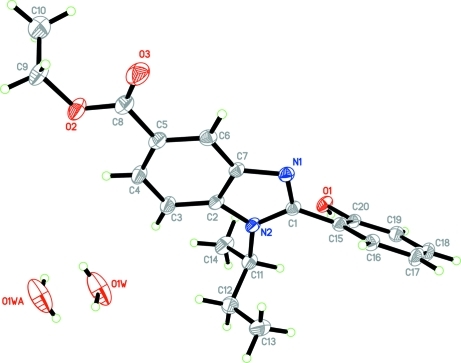
The asymmetric unit of the title compound, showing 50% probability displacement ellipsoids and the atom-numbering scheme.

**Fig. 2.**
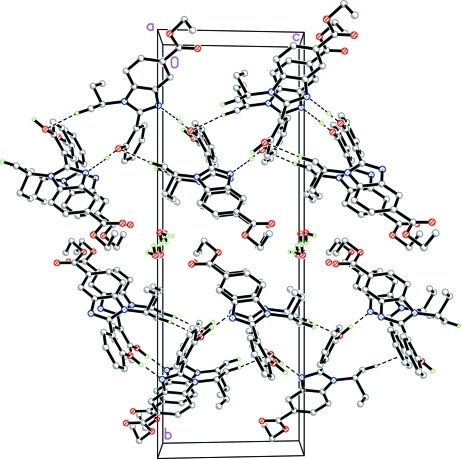
The crystal packing of the title compound, showing the hydrogen-bonded (dashed lines) network. H atoms not involved in hydrogen bond interactions are omitted for clarity.

## supplementary crystallographic information

### Comment

Benzimidazoles belong to one of the well known and most extensively studied class of  compounds due to their biological activity such as antitumour (Garuti *et al.*, 2004), antiviral (Bonfanti *et al.*, 2008), antibacterial (Ozden *et al.*, 2008) and analgesic properties (Shao *et al.*, 2005). These  derivatives are anti-inflammatory (Blythin *et al.*, 1986) and can be carcinogenic (Snow *et al.*, 2007). As the benzimidazole derivative is of much importance, we have undertaken the X-ray crystal structure determination of the title compound.

The asymmetric unit (Fig. 1) contains an ethyl-1-*sec*-butyl-2- (2-hydroxyphenyl)-1*H*-benzimidazole-5-carboxylate molecule and a water molecule(O1W), occupancy 0.25, which is disordered across a crystallographic inversion center (symmetry code = -x, -y+2, -z+1). The dihedral angle between the benzimidazole ring system (N1–N2/C1–C7) and the phenyl ring (C15–C20) is 59.31 (9)° .

In the crystal structure (Fig. 2), molecules are connected by intermolecular O1—H1O1···N1 and C14—H14C···O1 (Table 1) hydrogen bonds. The crystal structure is further stabilized by C—H···π interactions (Table 1), involving the imidazole ring, N1–N2/C1–C2/C7 (centroid Cg1).

### Experimental

The title compound was synthesised according to the previous procedure described by us (Arumugam *et al.*, 2010*a,b,c*). The product was recrystallized from EtOAc to yield the title compound as colourless crystals.

### Refinement

All hydrogen atoms were positioned geometrically [C–H = 0.93 or 0.97Å] and were refined using a riding model, with *U*_iso_(H) = 1.2 or 1.5 *U*_eq_(C). A rotating group model was applied to the methyl groups. In the final refinement cycles the occupancy of the water molecule, O1W, which is disordered over a crystallographic inversion centre, was fixed at 25%.

### Crystal data

**Table:**

C_20_H_22_N_2_O_3_·0.25H_2_O	*F*(000) = 730
*M**_r_* = 342.90	*D*_x_ = 1.263 Mg m^−^^3^
Monoclinic, *P*2_1_/*c*	Mo *K*α radiation, λ = 0.71073 Å
Hall symbol: -P 2ybc	Cell parameters from 3454 reflections
*a* = 7.0484 (11) Å	θ = 2.6–28.6°
*b* = 27.262 (4) Å	µ = 0.09 mm^−^^1^
*c* = 9.4673 (14) Å	*T* = 100 K
β = 97.495 (3)°	Plate, colourless
*V* = 1803.6 (5) Å^3^	0.34 × 0.21 × 0.05 mm
*Z* = 4	

### Data collection

**Table:**

Bruker APEX DUO CCD area-detector diffractometer	4738 independent reflections
Radiation source: fine-focus sealed tube	3152 reflections with *I* > 2σ(*I*)
graphite	*R*_int_ = 0.064
φ and ω scans	θ_max_ = 29.0°, θ_min_ = 2.3°
Absorption correction: multi-scan (*SADABS*; Bruker, 2009)	*h* = −9→9
*T*_min_ = 0.971, *T*_max_ = 0.996	*k* = −37→36
18277 measured reflections	*l* = −12→12

### Refinement

**Table:**

Refinement on *F*^2^	Primary atom site location: structure-invariant direct methods
Least-squares matrix: full	Secondary atom site location: difference Fourier map
*R*[*F*^2^ > 2σ(*F*^2^)] = 0.066	Hydrogen site location: inferred from neighbouring sites
*wR*(*F*^2^) = 0.232	H atoms treated by a mixture of independent and constrained refinement
*S* = 1.08	*w* = 1/[σ^2^(*F*_o_^2^) + (0.1283*P*)^2^ + 0.731*P*] where *P* = (*F*_o_^2^ + 2*F*_c_^2^)/3
4738 reflections	(Δ/σ)_max_ < 0.001
243 parameters	Δρ_max_ = 0.35 e Å^−^^3^
0 restraints	Δρ_min_ = −0.46 e Å^−^^3^

### Special details

**Table:**

Experimental. The crystal was placed in the cold stream of an Oxford Cryosystems Cobra open-flow nitrogen cryostat (Cosier & Glazer, 1986) operating at 100.0 (1) K.
Geometry. All s.u.'s (except the s.u. in the dihedral angle between two l.s. planes) are estimated using the full covariance matrix. The cell s.u.'s are taken into account individually in the estimation of s.u.'s in distances, angles and torsion angles; correlations between s.u.'s in cell parameters are only used when they are defined by crystal symmetry. An approximate (isotropic) treatment of cell s.u.'s is used for estimating s.u.'s involving l.s. planes.
Refinement. Refinement of F^2^ against ALL reflections. The weighted R-factor wR and goodness of fit S are based on F^2^, conventional R-factors R are based on F, with F set to zero for negative F^2^. The threshold expression of F^2^ > 2σ(F^2^) is used only for calculating R-factors(gt) etc. and is not relevant to the choice of reflections for refinement. R-factors based on F^2^ are statistically about twice as large as those based on F, and R- factors based on ALL data will be even larger.

### Fractional atomic coordinates and isotropic or equivalent isotropic displacement parameters (Å^2^)

**Table:**

	*x*	*y*	*z*	*U*_iso_*/*U*_eq_	Occ. (<1)
O1	0.3724 (2)	0.72773 (6)	0.28243 (18)	0.0229 (4)	
O2	−0.2905 (3)	0.97944 (7)	−0.0919 (2)	0.0330 (5)	
O3	−0.1402 (3)	0.95244 (8)	−0.2726 (2)	0.0416 (5)	
N1	0.3512 (3)	0.83123 (7)	0.0078 (2)	0.0194 (4)	
N2	0.3227 (3)	0.83949 (7)	0.2392 (2)	0.0198 (4)	
C1	0.4138 (3)	0.81661 (8)	0.1391 (2)	0.0185 (4)	
C2	0.1912 (3)	0.87134 (8)	0.1673 (2)	0.0200 (5)	
C3	0.0594 (3)	0.90402 (10)	0.2134 (3)	0.0257 (5)	
H3A	0.0478	0.9080	0.3095	0.031*	
C4	−0.0530 (3)	0.93018 (9)	0.1083 (3)	0.0258 (5)	
H4A	−0.1424	0.9523	0.1349	0.031*	
C5	−0.0366 (3)	0.92453 (9)	−0.0362 (3)	0.0243 (5)	
C6	0.0965 (3)	0.89227 (9)	−0.0812 (3)	0.0220 (5)	
H6A	0.1090	0.8886	−0.1772	0.026*	
C7	0.2102 (3)	0.86571 (8)	0.0232 (2)	0.0191 (5)	
C8	−0.1585 (4)	0.95277 (10)	−0.1479 (3)	0.0284 (5)	
C9	−0.4223 (4)	1.00752 (11)	−0.1914 (3)	0.0356 (6)	
H9A	−0.3573	1.0187	−0.2697	0.043*	
H9B	−0.4652	1.0362	−0.1437	0.043*	
C10	−0.5894 (4)	0.97724 (12)	−0.2473 (4)	0.0468 (8)	
H10A	−0.6797	0.9970	−0.3072	0.070*	
H10B	−0.6490	0.9646	−0.1694	0.070*	
H10C	−0.5481	0.9505	−0.3018	0.070*	
C11	0.3656 (3)	0.83308 (9)	0.3956 (2)	0.0231 (5)	
H11A	0.4583	0.8063	0.4127	0.028*	
C12	0.4596 (4)	0.87887 (10)	0.4644 (3)	0.0286 (5)	
H12A	0.3742	0.9066	0.4430	0.034*	
H12B	0.4790	0.8745	0.5669	0.034*	
C13	0.6503 (4)	0.89027 (11)	0.4137 (3)	0.0350 (6)	
H13A	0.7075	0.9181	0.4648	0.053*	
H13B	0.6305	0.8975	0.3136	0.053*	
H13C	0.7336	0.8624	0.4305	0.053*	
C14	0.1872 (3)	0.81788 (11)	0.4596 (3)	0.0314 (6)	
H14A	0.1249	0.7914	0.4050	0.047*	
H14B	0.1013	0.8453	0.4580	0.047*	
H14C	0.2228	0.8074	0.5562	0.047*	
C15	0.5701 (3)	0.78084 (8)	0.1704 (2)	0.0187 (5)	
C16	0.7463 (3)	0.79107 (9)	0.1229 (3)	0.0217 (5)	
H16A	0.7627	0.8203	0.0753	0.026*	
C17	0.8956 (3)	0.75806 (10)	0.1464 (3)	0.0262 (5)	
H17A	1.0132	0.7653	0.1167	0.031*	
C18	0.8696 (3)	0.71439 (10)	0.2137 (3)	0.0259 (5)	
H18A	0.9697	0.6920	0.2282	0.031*	
C19	0.6961 (3)	0.70333 (9)	0.2606 (3)	0.0228 (5)	
H19A	0.6800	0.6736	0.3056	0.027*	
C20	0.5463 (3)	0.73679 (9)	0.2401 (2)	0.0195 (5)	
H1O1	0.382 (5)	0.7052 (13)	0.361 (4)	0.048 (10)*	
O1W	0.0628 (16)	0.9740 (3)	0.4867 (10)	0.055 (3)	0.25
H1W1	−0.0325	0.9804	0.4320	0.083*	0.25
H2W1	0.1385	0.9962	0.4785	0.083*	0.25

### Atomic displacement parameters (Å^2^)

**Table:**

	*U*^11^	*U*^22^	*U*^33^	*U*^12^	*U*^13^	*U*^23^
O1	0.0148 (7)	0.0317 (9)	0.0230 (9)	−0.0007 (6)	0.0046 (6)	0.0064 (7)
O2	0.0285 (9)	0.0373 (11)	0.0324 (10)	0.0122 (8)	0.0006 (8)	0.0084 (8)
O3	0.0423 (12)	0.0533 (13)	0.0275 (11)	0.0139 (10)	−0.0021 (9)	0.0073 (9)
N1	0.0160 (8)	0.0256 (10)	0.0167 (9)	−0.0001 (7)	0.0026 (7)	−0.0001 (7)
N2	0.0157 (8)	0.0273 (10)	0.0164 (9)	0.0032 (7)	0.0013 (7)	0.0018 (7)
C1	0.0149 (9)	0.0238 (11)	0.0169 (11)	−0.0005 (8)	0.0024 (7)	0.0014 (8)
C2	0.0156 (10)	0.0261 (12)	0.0180 (11)	0.0011 (8)	0.0010 (8)	0.0021 (8)
C3	0.0202 (11)	0.0323 (13)	0.0247 (12)	0.0062 (9)	0.0039 (9)	0.0006 (10)
C4	0.0194 (11)	0.0302 (13)	0.0281 (13)	0.0060 (9)	0.0034 (9)	0.0020 (10)
C5	0.0211 (11)	0.0251 (12)	0.0255 (12)	0.0014 (9)	−0.0016 (9)	0.0034 (9)
C6	0.0222 (11)	0.0259 (12)	0.0175 (11)	−0.0005 (9)	0.0012 (8)	0.0013 (9)
C7	0.0152 (9)	0.0223 (11)	0.0197 (11)	−0.0006 (8)	0.0013 (8)	0.0006 (8)
C8	0.0259 (12)	0.0295 (13)	0.0285 (13)	0.0034 (10)	−0.0012 (10)	0.0046 (10)
C9	0.0289 (13)	0.0371 (15)	0.0392 (16)	0.0106 (11)	−0.0018 (11)	0.0118 (12)
C10	0.0392 (16)	0.0443 (18)	0.054 (2)	0.0057 (13)	−0.0043 (14)	−0.0063 (15)
C11	0.0211 (10)	0.0331 (13)	0.0153 (11)	0.0045 (9)	0.0034 (8)	0.0030 (9)
C12	0.0278 (12)	0.0352 (14)	0.0226 (12)	0.0057 (10)	0.0020 (9)	−0.0015 (10)
C13	0.0280 (13)	0.0375 (15)	0.0375 (16)	−0.0050 (11)	−0.0034 (11)	−0.0039 (12)
C14	0.0234 (12)	0.0494 (16)	0.0225 (13)	0.0021 (11)	0.0066 (9)	0.0076 (11)
C15	0.0164 (10)	0.0239 (11)	0.0157 (10)	−0.0001 (8)	0.0019 (8)	−0.0010 (8)
C16	0.0177 (10)	0.0280 (12)	0.0200 (11)	−0.0010 (9)	0.0050 (8)	0.0018 (9)
C17	0.0162 (10)	0.0388 (14)	0.0245 (12)	0.0029 (9)	0.0060 (9)	−0.0011 (10)
C18	0.0201 (11)	0.0334 (13)	0.0245 (12)	0.0069 (9)	0.0042 (9)	0.0005 (10)
C19	0.0228 (11)	0.0267 (12)	0.0189 (11)	0.0025 (9)	0.0026 (8)	0.0011 (9)
C20	0.0151 (10)	0.0279 (12)	0.0157 (10)	−0.0006 (8)	0.0030 (7)	−0.0011 (8)
O1W	0.099 (8)	0.032 (4)	0.046 (5)	−0.004 (5)	0.052 (6)	0.000 (4)

### Geometric parameters (Å, °)

**Table:**

O1—C20	1.361 (3)	C10—H10C	0.9600
O1—H1O1	0.96 (4)	C11—C12	1.519 (4)
O2—C8	1.343 (3)	C11—C14	1.523 (3)
O2—C9	1.452 (3)	C11—H11A	0.9800
O3—C8	1.204 (3)	C12—C13	1.518 (4)
N1—C1	1.325 (3)	C12—H12A	0.9700
N1—C7	1.389 (3)	C12—H12B	0.9700
N2—C1	1.363 (3)	C13—H13A	0.9600
N2—C2	1.383 (3)	C13—H13B	0.9600
N2—C11	1.483 (3)	C13—H13C	0.9600
C1—C15	1.472 (3)	C14—H14A	0.9600
C2—C7	1.396 (3)	C14—H14B	0.9600
C2—C3	1.397 (3)	C14—H14C	0.9600
C3—C4	1.386 (3)	C15—C20	1.391 (3)
C3—H3A	0.9300	C15—C16	1.403 (3)
C4—C5	1.396 (4)	C16—C17	1.380 (3)
C4—H4A	0.9300	C16—H16A	0.9300
C5—C6	1.393 (3)	C17—C18	1.374 (4)
C5—C8	1.487 (3)	C17—H17A	0.9300
C6—C7	1.392 (3)	C18—C19	1.388 (3)
C6—H6A	0.9300	C18—H18A	0.9300
C9—C10	1.479 (4)	C19—C20	1.389 (3)
C9—H9A	0.9700	C19—H19A	0.9300
C9—H9B	0.9700	O1W—O1W^i^	1.708 (18)
C10—H10A	0.9600	O1W—H1W1	0.8114
C10—H10B	0.9600	O1W—H2W1	0.8187
			
C20—O1—H1O1	112 (2)	C12—C11—C14	112.9 (2)
C8—O2—C9	116.6 (2)	N2—C11—H11A	107.4
C1—N1—C7	105.09 (19)	C12—C11—H11A	107.4
C1—N2—C2	106.95 (19)	C14—C11—H11A	107.4
C1—N2—C11	125.96 (18)	C13—C12—C11	112.8 (2)
C2—N2—C11	127.02 (19)	C13—C12—H12A	109.0
N1—C1—N2	112.65 (19)	C11—C12—H12A	109.0
N1—C1—C15	122.5 (2)	C13—C12—H12B	109.0
N2—C1—C15	124.82 (19)	C11—C12—H12B	109.0
N2—C2—C7	105.54 (19)	H12A—C12—H12B	107.8
N2—C2—C3	132.6 (2)	C12—C13—H13A	109.5
C7—C2—C3	121.9 (2)	C12—C13—H13B	109.5
C4—C3—C2	116.4 (2)	H13A—C13—H13B	109.5
C4—C3—H3A	121.8	C12—C13—H13C	109.5
C2—C3—H3A	121.8	H13A—C13—H13C	109.5
C3—C4—C5	122.3 (2)	H13B—C13—H13C	109.5
C3—C4—H4A	118.8	C11—C14—H14A	109.5
C5—C4—H4A	118.8	C11—C14—H14B	109.5
C6—C5—C4	120.9 (2)	H14A—C14—H14B	109.5
C6—C5—C8	117.4 (2)	C11—C14—H14C	109.5
C4—C5—C8	121.7 (2)	H14A—C14—H14C	109.5
C7—C6—C5	117.4 (2)	H14B—C14—H14C	109.5
C7—C6—H6A	121.3	C20—C15—C16	119.4 (2)
C5—C6—H6A	121.3	C20—C15—C1	122.27 (19)
N1—C7—C6	129.1 (2)	C16—C15—C1	118.3 (2)
N1—C7—C2	109.77 (19)	C17—C16—C15	120.5 (2)
C6—C7—C2	121.1 (2)	C17—C16—H16A	119.8
O3—C8—O2	124.0 (2)	C15—C16—H16A	119.8
O3—C8—C5	124.6 (2)	C18—C17—C16	119.6 (2)
O2—C8—C5	111.4 (2)	C18—C17—H17A	120.2
O2—C9—C10	110.5 (2)	C16—C17—H17A	120.2
O2—C9—H9A	109.5	C17—C18—C19	120.9 (2)
C10—C9—H9A	109.5	C17—C18—H18A	119.6
O2—C9—H9B	109.5	C19—C18—H18A	119.6
C10—C9—H9B	109.5	C18—C19—C20	119.9 (2)
H9A—C9—H9B	108.1	C18—C19—H19A	120.1
C9—C10—H10A	109.5	C20—C19—H19A	120.1
C9—C10—H10B	109.5	O1—C20—C19	122.5 (2)
H10A—C10—H10B	109.5	O1—C20—C15	117.73 (19)
C9—C10—H10C	109.5	C19—C20—C15	119.8 (2)
H10A—C10—H10C	109.5	O1W^i^—O1W—H1W1	60.9
H10B—C10—H10C	109.5	O1W^i^—O1W—H2W1	75.8
N2—C11—C12	110.6 (2)	H1W1—O1W—H2W1	106.0
N2—C11—C14	110.99 (19)		
			
C7—N1—C1—N2	−0.1 (2)	C6—C5—C8—O3	−5.9 (4)
C7—N1—C1—C15	−178.0 (2)	C4—C5—C8—O3	173.6 (3)
C2—N2—C1—N1	0.0 (3)	C6—C5—C8—O2	175.0 (2)
C11—N2—C1—N1	−176.9 (2)	C4—C5—C8—O2	−5.5 (3)
C2—N2—C1—C15	177.8 (2)	C8—O2—C9—C10	87.3 (3)
C11—N2—C1—C15	0.8 (4)	C1—N2—C11—C12	109.2 (2)
C1—N2—C2—C7	0.1 (2)	C2—N2—C11—C12	−67.2 (3)
C11—N2—C2—C7	177.0 (2)	C1—N2—C11—C14	−124.7 (2)
C1—N2—C2—C3	−179.9 (3)	C2—N2—C11—C14	59.0 (3)
C11—N2—C2—C3	−2.9 (4)	N2—C11—C12—C13	−62.2 (3)
N2—C2—C3—C4	−179.3 (2)	C14—C11—C12—C13	172.8 (2)
C7—C2—C3—C4	0.7 (4)	N1—C1—C15—C20	−121.4 (2)
C2—C3—C4—C5	−0.1 (4)	N2—C1—C15—C20	61.0 (3)
C3—C4—C5—C6	−0.6 (4)	N1—C1—C15—C16	56.0 (3)
C3—C4—C5—C8	179.9 (2)	N2—C1—C15—C16	−121.5 (2)
C4—C5—C6—C7	0.7 (3)	C20—C15—C16—C17	−0.7 (3)
C8—C5—C6—C7	−179.8 (2)	C1—C15—C16—C17	−178.3 (2)
C1—N1—C7—C6	−179.3 (2)	C15—C16—C17—C18	1.5 (4)
C1—N1—C7—C2	0.2 (2)	C16—C17—C18—C19	−1.0 (4)
C5—C6—C7—N1	179.4 (2)	C17—C18—C19—C20	−0.4 (4)
C5—C6—C7—C2	−0.1 (3)	C18—C19—C20—O1	179.3 (2)
N2—C2—C7—N1	−0.2 (2)	C18—C19—C20—C15	1.2 (4)
C3—C2—C7—N1	179.8 (2)	C16—C15—C20—O1	−178.9 (2)
N2—C2—C7—C6	179.3 (2)	C1—C15—C20—O1	−1.4 (3)
C3—C2—C7—C6	−0.7 (4)	C16—C15—C20—C19	−0.6 (3)
C9—O2—C8—O3	2.8 (4)	C1—C15—C20—C19	176.8 (2)
C9—O2—C8—C5	−178.2 (2)		

Symmetry codes: (i) −*x*, −*y*+2, −*z*+1.

### Hydrogen-bond geometry (Å, °)

**Table:**

Cg1 is the centroid of the N1,N2,C1,C2,N7 imidazole ring.

**Table:**

*D*—H···*A*	*D*—H	H···*A*	*D*···*A*	*D*—H···*A*
O1—H1O1···N1^ii^	0.96 (4)	1.75 (4)	2.691 (3)	168 (3)
C14—H14C···O1^ii^	0.96	2.45	3.398 (3)	168
C17—H17A···Cg1^iii^	0.93	2.96	3.734 (3)	142

Symmetry codes: (ii) *x*, −*y*+3/2, *z*+1/2; (iii) *x*+1, *y*, *z*.
